# Computing with Residue Numbers in High-Dimensional Representation

**Published:** 2023-11-08

**Authors:** Christopher J. Kymn, Denis Kleyko, E. Paxon Frady, Connor Bybee, Pentti Kanerva, Friedrich T. Sommer, Bruno A. Olshausen

**Affiliations:** 1 Redwood Center for Theoretical Neuroscience, University of California, Berkeley, CA; 2 Centre for Applied Autonomous Sensor Systems, Örebro University, Sweden; 3 Intelligent Systems Lab, Research Institutes of Sweden, Kista, Sweden; 4 Neuromorphic Computing Lab, Intel, Santa Clara, CA

## Abstract

We introduce *Residue Hyperdimensional Computing*, a computing framework that unifies residue number systems with an algebra defined over random, high-dimensional vectors. We show how residue numbers can be represented as high-dimensional vectors in a manner that allows algebraic operations to be performed with component-wise, parallelizable operations on the vector elements. The resulting framework, when combined with an efficient method for factorizing high-dimensional vectors, can represent and operate on numerical values over a large dynamic range using vastly fewer resources than previous methods, and it exhibits impressive robustness to noise. We demonstrate the potential for this framework to solve computationally difficult problems in visual perception and combinatorial optimization, showing improvement over baseline methods. More broadly, the framework provides a possible account for the computational operations of grid cells in the brain, and it suggests new machine learning architectures for representing and manipulating numerical data.

## 1 Introduction

The problem of representing and computing on high-dimensional representations of numerical values—such as position, velocity, and color—is central to both machine learning and computational neuroscience. In machine learning, vector representations of numbers are useful for defining position or function encodings in neural networks [1]–[3], improving robustness to adversarial examples [4], and generating efficient classifiers [5]. In neuroscience, experimentalists seek to understand how populations of neurons in the brain represent and transform perceptual or cognitive variables, and so numerous theorists have constructed models for how these variables could be encoded in and decoded from high-dimensional vector encodings (e.g., [6]–[8]).

A particularly salient example of high-dimensional representation in neuroscience is the ‘grid cell’ encoding of spatial position in the medial entorhinal cortex [9]. Grid cells have multiple peaks in their firing rates that correlate with spatial positions arranged in a hexagonal lattice. While somewhat perplexing at first glance, the usefulness of such a coding scheme becomes apparent from how it functions as a population code. In comparison to a population of neurons with traditional unimodal encoding functions whose coding resolution increases linearly with the number of neurons, a grid cell population possesses a coding resolution that grows exponentially in the number of neurons [10]. In particular, Fiete and colleagues have emphasized that this advantage of grid cell encoding utilizes properties of *residue numbers* (see Section 2.2 below) [11].

Inspired by this observation, we propose a comprehensive algebraic framework for distributed neural computation based on residue number systems. Our novel framework builds on an existing algebraic framework for computing with high-dimensional random vectors. The idea was originally called computing in Holographic Reduced Representation [12] and is now referred to also as *Vector Symbolic Architectures* (VSA) [13] and *Hyperdimensional Computing* [14]. We call our new framework Residue *Hyperdimensional Computing* (RHC) and demonstrate that it inherits the computational advantages of both standard residue number systems and hyperdimensional computing. This enables fault-tolerant computations that can efficiently represent numbers and search over a large dynamic range with greatly reduced memory requirements. Furthermore, as we shall see, the new framework provides a useful formalism for understanding computations in grid cells.

To summarize, we list the four key coding properties we achieve with Residue Hyperdimensional Computing: (1) Algebraic structure: Simple operations on vectors perform addition and multiplication on encoded values, (2) Expressivity: Feasible encoding range scales better than linearly with dimension, (3) Efficient decoding: Required resources to decode scale better than linearly with encoding range, and (4) Robustness to noise. Although a number of previously proposed models achieve some of these properties (Table S.1, Supplement (A), Residue Hyperdimensional Computing is the first, to our knowledge, to *achieve all four of these desiderata*, as we shall now show.

## 2 Results

We first define the key concepts on which the Residue Hyperdimensional Computing framework is based (Section 2.1) and then describe the framework fully in Section 2.2. We then demonstrate its favorable encoding, decoding, and robustness properties (Section 2.3), as well as how it can be extended to multiple dimensions (Section 2.4) and sub-integer encodings (Section 2.5). Of particular note, we construct a hexagonal residue encoding system, analogous to grid cell coordinates, that provides higher spatial resolution than square lattices. Finally, we describe how the framework can be applied to problems in visual scene analysis and combinatorial optimization (Section 2.6).

### 2.1 Preliminary definitions

#### Definition 2.1.1.

**A Residue Number System** [15] encodes an integer x∈Z by its value modulo $\left\{m_{1},m_{2},\ldots ,m_{K}\right\}$, where the $m_{k}$ are the *moduli* of the system. For example, relative to moduli {3, 5, 7}, x=20 would be encoded by the residue [2, 0, 6]—i.e., [20 mod 3, 20 mod 5, 20 mod 7]. The Chinese Remainder Theorem states that if the moduli are pairwise co-prime, then for any x such that $0\leq x<M:=\prod_{k}\;m_{k}$, the integer is uniquely encoded by its residue [16]. From here on, we will assume that the pairwise co-prime condition is fulfilled.

#### Definition 2.1.2.

**Fractional Power Encoding (FPE**) [12] defines a randomized mapping from an integer x to a high-dimensional vector z(x). Let D be the dimension of the vector, with D typically in the range $10^{2}\leq D\leq 10^{4}$. The encoding scheme involves two steps. First, draw a random base vector, z, defined as follows:

(1) $$z=\left[e^{i\phi_{1}},e^{i\phi_{2}},\ldots ,e^{i\phi_{D}}\right]$$

where each element $e^{i\phi_{j}}$ is a complex number with unit magnitude (a *phasor*), and each $\phi_{j}$ is a random sample from a specified probability distribution. Second, define a function from x to $C^{D}$ via component-wise exponentiation of the random vector:

(2) $$z(x)=z^{x}=\left[e^{i\phi_{1}x},e^{i\phi_{2}x},\ldots ,e^{i\phi_{D}x}\right]$$

#### Definition 2.1.3.

A **kernel**, $K\left(x_{1},x_{2}\right)$, is a function χ×χ→R that measures the similarity between two objects in a set χ (e.g., vectors in $R^{n}$). Notably, FPE implements kernel approximation [17], which is widely used in machine learning [18]. More specifically, we can induce a kernel based on the inner products of FPEs:

(3) $$K\left(x_{1},x_{2}\right)=\frac{1}{D}R\left\{z{\left(x_{1}\right)}^{T}\overset{-}{z\left(x_{2}\right)}\right\}$$

where $\overset{-}{z\left(x_{2}\right)}$ is the complex conjugate of $z\left(x_{2}\right)$. This defines a translation-invariant kernel K(Δx) (where $\Delta x=x_{1}-x_{2}$), which converges to a particular $K^{*}(\Delta x)$ as D→∞, where the shape of the kernel is determined by the probability distribution used to draw z [19], [20].

### 2.2 Residue Hyperdimensional Computing

We now introduce how FPE can implement a residue number system. As a first step, we explain how FPE can implement *congruence* (representing a remainder, modulo m).

#### Definition 2.2.1.

**Fractional Power Encoding, modulo** m: For $z_{m}$, let the support of our probability distribution for the $\phi_{j}$ be the m-th roots of unity. In other words, each $\phi_{j}$ must be a multiple of 2π/m. Then congruent values are mapped to the same vector:

$$z_{m}\left(x+m\right)=\left[e^{i\phi_{1}\left(x+m\right)},e^{i\phi_{2}\left(x+m\right)},\ldots ,e^{i\phi_{D}\left(x+m\right)}\right]\;=\left[e^{i\phi_{1}x}\cdot e^{i\phi_{1}m},e^{i\phi_{2}x}\cdot e^{i\phi_{2}m},\ldots ,e^{i\phi_{D}x}\cdot e^{i\phi_{D}m}\right]\;=\left[e^{i\phi_{1}x},e^{i\phi_{2}x},\ldots ,e^{i\phi_{D}x}\right]\;=z_{m}\left(x\right)$$

because $e^{i\phi_{j}m}=e^{2\pi \cdot i\cdot k}$ for some integer k, and $e^{2\pi \cdot i\cdot k}=1$ for any integer k. Put another way, $z_{m}(x)$ is a *representation* (in the abstract algebraic sense) of the additive group of integers modulo m.

**Figure 1:**
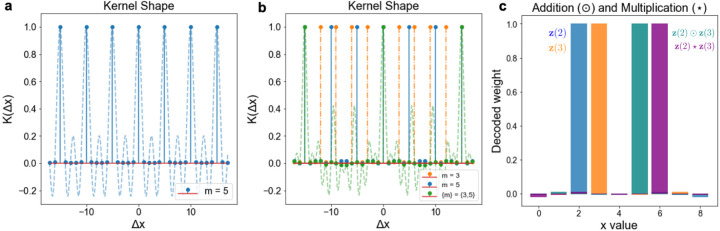
Residue Hyperdimensional Computing defines a kernel separating different remainder values, and it enables algebraic operations. **a**, For fractional power encoding, modulo m=5, inner products between vectors reflect the similarity of points with the same remainder value and are quasi-orthogonal elsewhere. The light blue curve shows the kernel shape when Δx is a real-valued scalar; integers occur approximately at zero crossings. A further derivation is provided in Supplement B. **b**, The kernel induced by an RHC vector (green) is the product kernel of the moduli used to form it (orange, blue). **c**, Demonstration of addition and multiplication. Blue and orange show encodings of 2 and 3, respectively. Teal shows the decoded value of 2+3 (i.e., 5); purple shows decoded value of 2 × 3 (i.e., 6).

The kernel induced by $z_{m}(\Delta x)$ is 1 if Δx=0(modm), and ≈ 0 otherwise, as shown in Figure 1a. This is highly useful, because it implies that distinct integers behave as quasi-orthogonal vectors, just like symbols in hyperdimensional computing. Unlike symbols, however, we can perform algebraic manipulations transforming one integer into another.

#### Definition 2.2.2.

**Residue Hyperdimensional Computing**: Let $z_{m_{1}},z_{m_{2}},\ldots ,z_{m_{K}}$ denote FPE vectors with moduli $m_{1},m_{2},\ldots ,m_{K}$ respectively. Let ⊙ denote component-wise multiplication (a.k.a. Hadamard product). Then, we encode an integer x by combining our modulo representations via the Hadamard product:

(4) $$z(x)=\overset{K}{\underset{k=1}{⨀}}\;z_{m_{k}}(x)$$

The above encoding represents the remainder of x, because each $z_{m_{k}}$ represents its value modulo $m_{k}$. The code is fully distributed, as every element of the vector z contains information about each encoding vector $z_{m_{k}}(x)$. By contrast, typical implementations of residue number systems compartmentalize information about individual moduli [21].

The kernel induced by z(Δx) is 1 if $\Delta x=0\left(\text{mod}\;\prod_{k}\;m_{k}=M\right)$, and ≈ 0 for other integer intervals Δx (Figure 1b). This means that the kernel maps different remainders of our residue number system to quasi-orthogonal directions in high-dimensional vector space, and enables computing in superposition over these variables. Examples of possible applications enabled by such a scheme are presented in Section 2.6.

The hallmark of a residue number system is *carry-free arithmetic*; that is, addition, subtraction, and multiplication can be performed component-wise on the remainders. This enables residue number systems to be highly parallel, avoiding carryover operations required in binary number systems. *Residue Hyperdimensional Computing implements arithmetic with component-wise operations, thus inheriting the premier computational property of residue number systems*.

Addition is defined as the Hadamard product between vectors, that is $z\left(x_{1}+x_{2}\right)=z\left(x_{1}\right)⊙z\left(x_{2}\right)$ (Methods 4.1.1). This follows from the fact that component-wise multiplication of phasors corresponds to phase addition, and that component-wise multiplication is commutative. Subtraction is defined by addition of the additive inverse.

Next, we define a second binding operation that implements multiplication, denoted as $⋆:z\left(x_{1}\cdot x_{2}\right)=z\left(x_{1}\right)⋆z\left(x_{2}\right)$. Just as variable addition is implemented by element-wise multiplication, variable multiplication is implemented by another element-wise operation, this one involving exponentiation (Methods 4.1.2). Here, it is crucial that our encoding function is restricted to integers as its domain, because multiplication is commutative and integer powers commute (that is, ${\left(c^{x_{1}}\right)}^{x_{2}}=c^{x_{1}x_{2}}={\left(c^{x_{2}}\right)}^{x_{1}}$ for c∈C and integers $x_{1},x_{2}$). We show how this definition for integer multiplication can be generalized to multiplication for vector encodings $z\left(x_{1}\right)$ and $z\left(x_{2}\right)$, without invoking the costly step of first decoding the integers back from the vectors.

Division is not well-defined for residue number systems, because integers are not closed under division. Still, when a modulus $m_{k}$ is prime, multiplications by non-zero integers are invertible, because each non-zero integer has a unique multiplicative inverse with respect to $m_{k}$.

*The existence of two distinct binding operators for addition and multiplication is a new contribution to hyperdimensional computing*. Previous formulations only supported addition, or only multiplication via addition after taking logarithmic transformations [22]. Consequently, we can now formulate a fully distributed residue number system that inherits the benefits of computing with high-dimensional vectors.

### 2.3 The resonator network enables efficient decoding of residue numbers

Given the vector representation of a residue number, z(x), how do we decode it to recover its value, x? One method for decoding commonly used in hyperdimensional computing is *codebook decoding* [23], which involves taking the inner product of z(x) with a set of M codebook vectors with known values of x. However, this procedure requires 𝒪(M*D) storage and M inner product evaluations.

**Figure 2:**
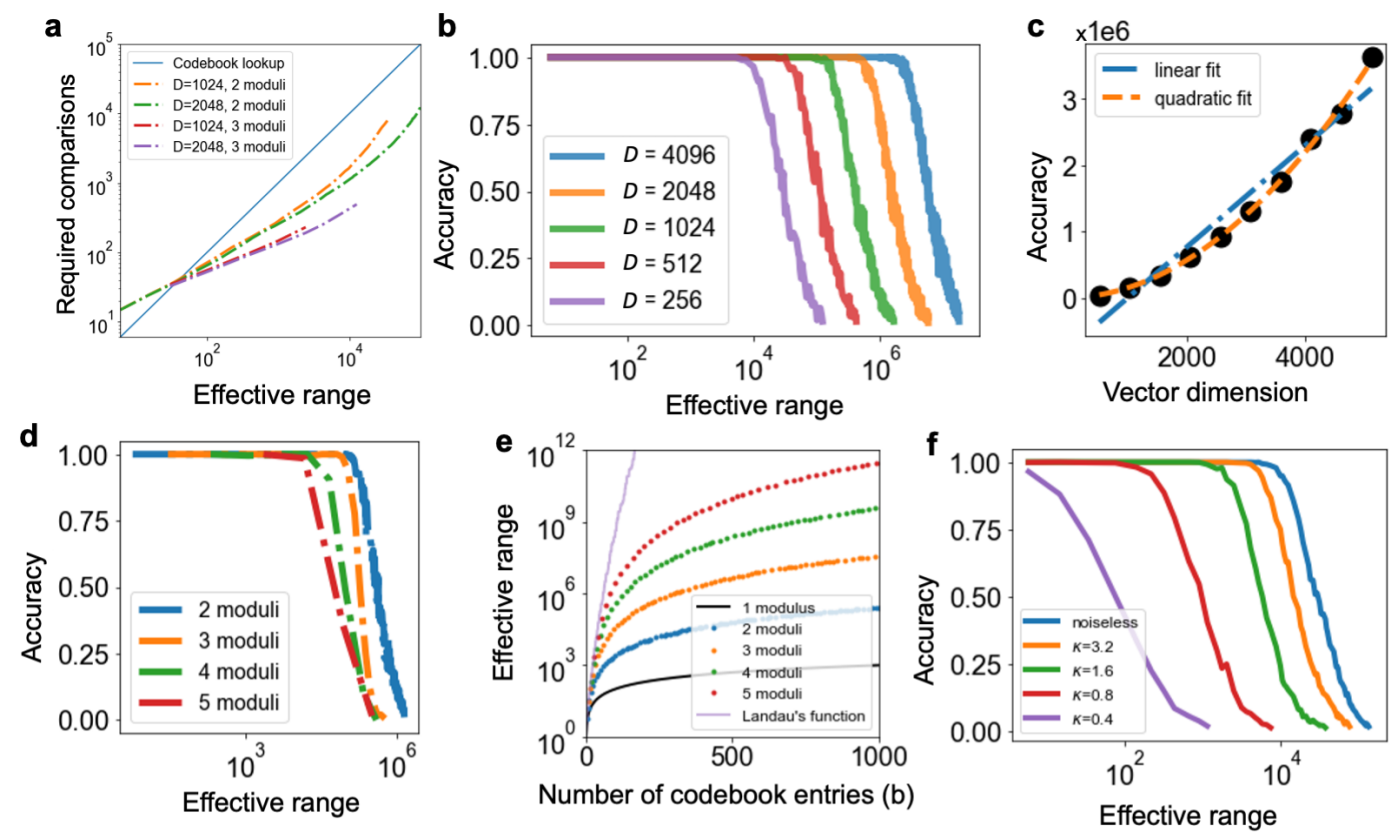
The resonator network can efficiently recover moduli of different numbers. **a**, The resonator network outperforms codebook decoding by over an order of magnitude when the effective range is within resonator network capacity. **b**, Demonstration of resonator network capacity. For a fixed vector dimension, accuracy remains high up to a given range, before gradually falling off. **c**, Scaling of resonator network capacity, C, as a function of dimension, D, is well-described by a quadratic fit (orange dashed line; cf. linear fit in blue dash-dotted line). Quadratic fit coefficients are $\left(1.3\times 10^{-1}\right)D^{2}+\left(2.4\times 10^{1}\right)D-3.7\times 10^{3}$, linear fit coefficients are $\left(7.6\times 10^{2}\right)D-7.6\times 10^{5}$. **d**, Resonator network performance is slightly worse for a higher number of moduli, K, but **e**, an advantage of lower moduli is a much higher effective range given encoding resources. **f**, The capacity of the resonator network remains high even in the presence of large amounts of phase noise.

Fortunately, we can improve the situation by utilizing the fact that residue numbers break the dynamic range of a number into a set of smaller numbers, each with lower overall dynamic range. For example, a number with dynamic range of 105, when represented modulo [3, 5, 7], consumes a total dynamic range of 3+5+7 = 15. This in turn reduces both the memory and computation resources for decoding by a factor of 105/15 = 7. To make this work though requires that we invert Equation 4—that is, we must factorize z(x) into the set of constituent vectors $\left\{z_{m_{1}}(x),z_{m_{2}}(x),\ldots ,z_{m_{K}}(x)\right\}$ representing x modulo $m_{k}$, from which x can be easily recovered. For this we can use a *resonator network* [24], [25], a recently discovered method for efficiently factorizing vectors in hyperdimensional computing. Figure 2a shows that for a range of M values, the resonator network can recover vectors over an order of magnitude faster than standard codebook decoding. Two parameters that contribute to this are the vector dimension (D) and number of moduli (K).

To evaluate the dependence of resonator decoding on vector dimension, we fix the number of moduli (K=2) and calculate the empirical accuracy (Figure 2b) of the resonator on the effective range M. We find that for a fixed D, the accuracy remains almost perfect up to a certain range of M, after which accuracy rapidly decays. To evaluate scaling with D, we define the capacity C of a dimension to be the largest M up to which empirical accuracy is at least 95 percent. We find that the scaling of C(D) is well-fit with a quadratic polynomial (Figure 2c), consistent with previous scaling laws studied for a resonator network with two states per component [25]. Further tests with higher dimensions would help confirm quadratic scaling, but even the linear scaling has high slope (and note that $C(4096)>\;\left.2\times 10^{6}\right)$.

To evaluate dependence on the number of moduli, we fix D=1024 and vary K. We find that resonator capacity decreases as K increases (Figure 2d), also consistent with prior work [25]. Still, we emphasize that resonators with higher K have two advantages: decreased computation per decoding (Figure 2a), and decreased memory requirements. The resonator requires only $\sum_{k}\;m_{k}=b$ codebook vectors, rather than $\prod_{k}\;m_{k}=M$. This means that increasing K can increase the effective range by *several orders of magnitude* given a fixed codebook budget (Figure 2e). Remarkably, the maximal M for a given b is given by Landau’s function g(b), which scales as $g(b)=e^{(1+o(1))\sqrt{b\text{ln}b}}$ [26]. This implies an exponential scaling between storage required and effective range, if we proffer sufficient K to achieve it.

Finally, we evaluate how robust resonator network decoding is to noise. We draw phase noise from a von Mises distribution with mean 0 and concentration κ; higher κ indicates less noise. In Figure 2f, we observe that performance degrades gradually as a function of noise, yet capacity remains remarkably high even at high noise levels.

### 2.4 Generalization to multiple dimensions

#### 2.4.1 Cartesian representations of $Z^{n}$

Next, we generalize Residue Hyperdimensional Computing from scalars to multi-dimensional variables, showing that the core operations and principles still apply. Let $x\in Z^{n}$ be a low-dimensional vector. Let $x_{1},x_{2},\ldots ,x_{n}$ denote the components of x. To encode x with a single high-dimensional vector $z\in V^{D}(D>>n)$ we form the encoding z(x) by taking the Hadamard product of the distributed representations of individual components:

(5) $$z(x)=z_{1}\left(x_{1}\right)⊙z_{2}\left(x_{2}\right)⊙\cdots ⊙z_{n}\left(x_{n}\right)$$

where each $z_{i}$ is a random vector as generated for the residue representation of a single number in Equation (4). (Each $z_{i}$ is created from binding multiple vectors for each of the moduli, distinct for each dimension i.) Since the binding operation is commutative, we can rearrange our terms to show the following useful properties hold:

- The Hadamard product (⊙) performs vector addition: $z(x)⊙z\left(x^{'}\right)=z\left(x+x^{'}\right)$.
- The multiplicative binding operation (⋆) performs component-wise multiplication of x and $x^{'}:z(x)⋆z\left(x^{'}\right)=z\left(x⊙x^{'}\right)$.
- The kernel induced by z is the product of the kernels of the individual components: $K_{z}(\Delta x)=\prod_{i=1}^{n}\;K_{z_{i}}\left(\Delta x_{i}\right)$, where $\Delta x=x-x^{'}$.

While (a) and (c) are general properties of FPE, (b) is once again unique to Residue Hyperdimensional Computing. In addition, the savings in decoding resources and computation required (Figure 2) also scale in higher dimensions, as utilized in Section 2.6.

#### 2.4.2 Hexagonal coordinate systems

When working in a multi-dimensional space, there are multiple alternatives to a Cartesian coordinate system. For example, grid cells in medial entorhinal cortex encode spatial location with a hexagonal coordinate system; research in theoretical neuroscience suggests that this is because such a tiling of space has the highest resolution (Fisher information) in 2D space [27]. As an illustrative example, we show that Residue Hyperdimensional Computing can also implement hexagonal coordinate systems, and that such a hexagonal lattice retains coding advantages over square lattices. Of particular note, we formulate a self-consistent encoding that extends a residue number system to non-negative hexagonal coordinates.

**Figure 3:**
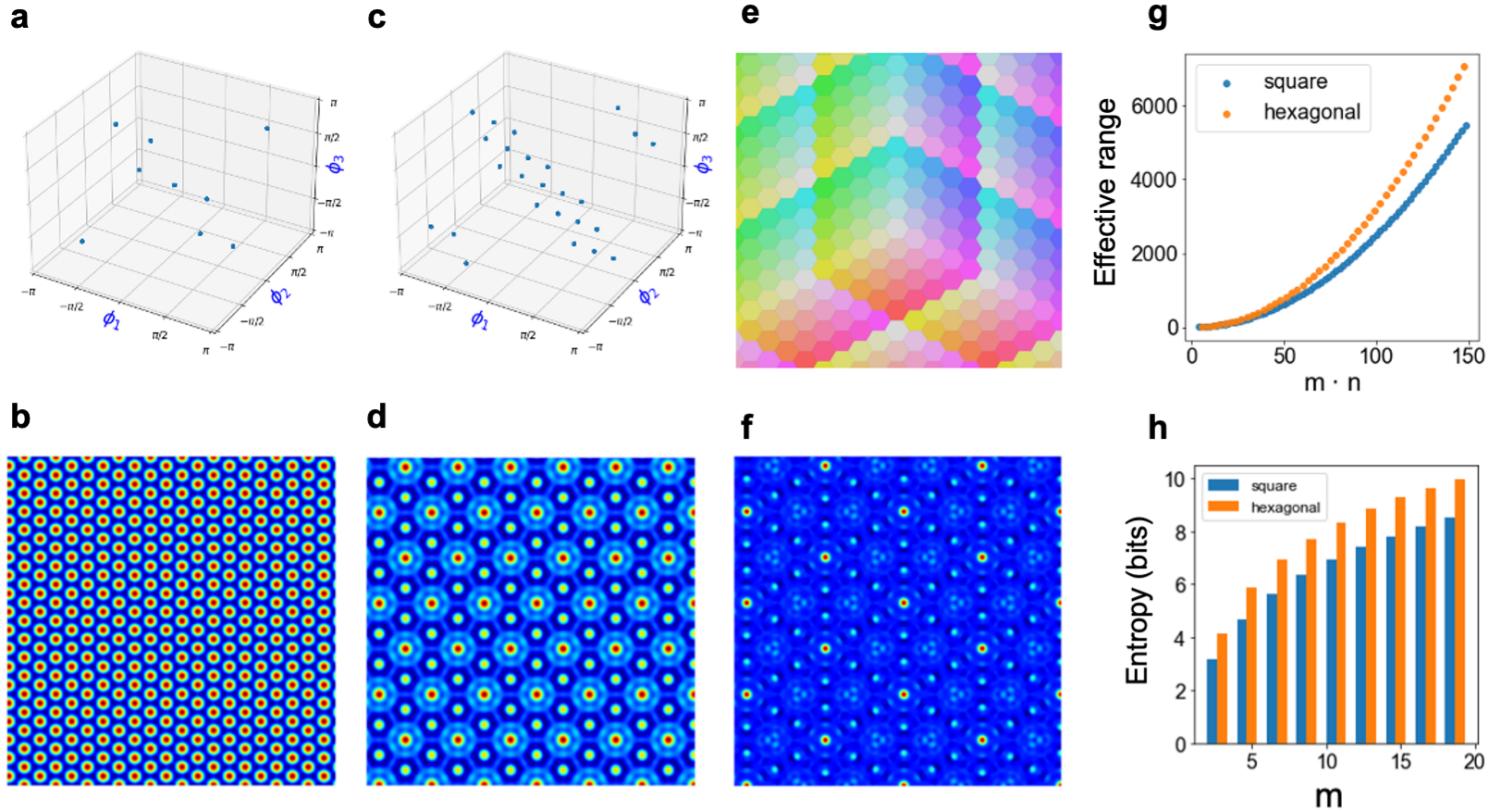
Definition of a residue number system in non-negative hexagonal coordinates. **a** and **c** show discrete phase distributions chosen for a 3D coordinate system of a 2D space with moduli 3 and 5, respectively. In addition to requiring that phases are drawn from the m-th roots of unity, we enforce that the three phases sum to 0 (mod2π). **b** and **d** show the respective kernels generated by these phase distributions. **e**, An example of the Voronoi tesselation of different states composed of a hexagonal coordinate system with modulus 5. Each color corresponds to a different state representation in the vector space of integers. **f**, The kernel induced by a hexagonal residue HD vector (period of 3 · 5 = 15). **g**, Compared to square encodings of space, hexagonal encodings approximately triple the effective range of encodable states with only a 50 percent increase in required storage space. **h**, The Shannon entropy of the hexagonal code is higher than that of the square code with the same modulus, reflecting the benefit of hexagonal packing.

To encode a two-dimensional position into a three-coordinate frame requires two steps. First, we project the 2D vector x into a 3D vector y with unit vectors whose angles each differ by $\frac{2\pi }{3}$ (Methods 4.3). This coordinate representation is known as the ‘Mercedes-Benz’ frame in $R^{2}$; it is well-studied in signal processing [28] and has attracted recent interest in hyperdimensional computing (e.g., [19], [29]). For our purposes, this step is necessary, but not sufficient, because projections can result in negative values. Second, to rectify values, we encode y with the method described in Section 2.4.1, but with the additional constraint that z([1,1,1])=z([0,0,0]), ensuring that every state with negative coordinates has an equivalent representation to one with non-negative coordinates (Figures 3a and 3c, Methods 4.3). This constraint also reflects the fact that equal movement in every direction cancels out, and thus it enforces that different paths to the same 2D position result in the same high-dimensional encoding. The kernels induced by vectors of individual moduli (Figures 3b and 3d) and by the residue vector (Figure 3f) exhibit the six-fold symmetry characteristic of hexagonal lattices and grid cells [9].

We can, therefore, represent the hexagonal coordinate system with a Voronoi tesselation (Figure 3e) in which different regions of space are mapped to their nearest integer-valued 3D coordinate. A hexagonal system with modulus m has $3m^{2}-3m+1$ distinct states and requires 3m codebook vectors, whereas a square lattice has $m^{2}$ distinct states and requires 2m codebook vectors (Figure 3g). Thus, the hexagonal system achieves better spatial resolution (it is a higher entropy code with regards to space) than a square lattice (Figure 3h) for the same number of resources.

### 2.5 Extensions to sub-integer decoding resolution

**Figure 4:**
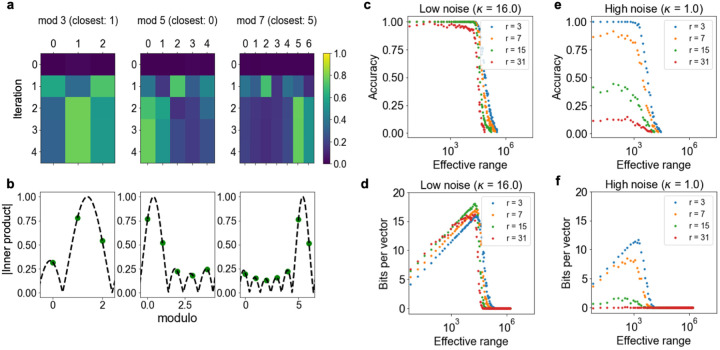
The resonator network enables retrieval of fractional (sub-integer) values. **a**, Example of the resonator network converging for z(x),x=40.4. **b**, Inner product values decoded by the resonator network are predicted by fractional offsets from a Dirac comb convolved with a sinc function. **c**, Sub-integer encoding accuracy under a low noise regime for a RHC vector (r denotes number of sub-integer partitions). **d**, Bits per vector for different fractional decodings. **e**, **f**, same as **c** and **d**, respectively, but under higher noise conditions.

In previous sections, we worked exclusively with integer states and residue number systems implementing them. Intriguingly, however, we can extend our definition of FPE to rational numbers (Methods 4.4.1), and the resonator converges to FPE encodings of non-integers, even when codebooks contain only encodings of integers (Figures 4a and b). Strictly speaking, such extensions beyond integers are not residue number systems, and multiplicative binding is no longer well-defined. However, extensions towards sub-integer resolution have been considered in theoretical analyses of grid cells, e.g., in [11], [30], and we show that the resonator dynamics achieve this sub-integer resolution.

An efficient procedure for decoding with sub-integer precision is suggested by Figure 4b. The inner products between codebook states and the final resonator network state are well-described by evaluations of a Dirac comb convolved with a sinc function (Supplement B). For integer encodings, this function would evaluate to 0 for all features not near the peak, but for non-integers this is no longer the case. Still, we can find the sub-integer offset that best matches the resonator network state in order to decode the sub-integer value (Methods 4.4.2).

Phase noise is the limiting factor in decoding sub-integer states. To quantify this more rigorously, we evaluate a resonator network with varying effective ranges M under different noise regimes (κ=16 and 1, respectively). We then split each unit interval into r partitions, so that there are M·r distinct numbers represented. Figures 4c and 4e show the accuracy of decoding for a different number of partitions with κ=16 and κ=1, respectively. To account for accuracy and the number of different states distinguished, we also report the ‘bits per vector’ metric [31] in Figures 4d and 4f. This metric validates that with lower noise, we can more reliably decode a higher number of states.

### 2.6 Applications

#### 2.6.1 Efficient disentangling of object shape and pose from images

Here we study the disentangling problem in vision—that is, the task of recovering the underlying components of a scene given only the image pixel values. Such problems abound in visual perception; examples include inferring structure-from-motion or separating spectral reflectance properties from shading due to 3D shape and lighting. How brains disentangle these factors of variation in general is unknown, and it is computationally challenging due to the combinatorial explosion in how factors combine to create any given scene [32].

**Figure 5:**
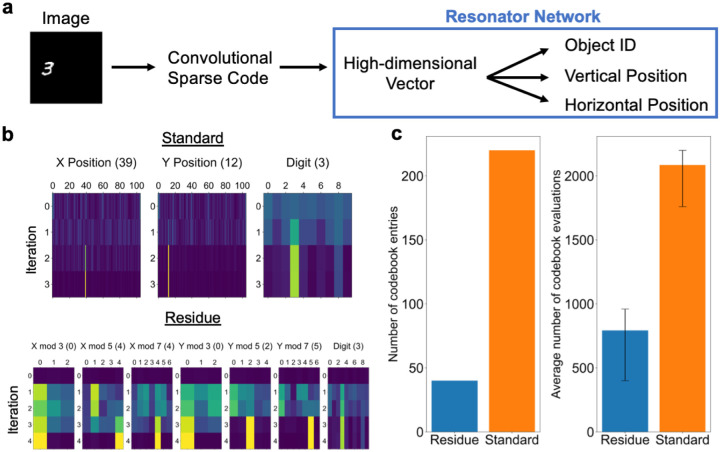
Residue Hyperdimensional Computing enables efficient disentangling of images. **a**, Processing pipeline: an image is first represented in terms of its local shape features via convolutional sparse coding, then converted to a high-dimensional vector z by superimposing the residue number encodings of the positions of each feature in the image, which is finally factorized into object identity and position via a resonator network. **b**, Simulations of the resonator network on visual scenes without the residue number encoding (Standard) and with the residue number encoding (Residue). **c**, With a residue number system, the resonator network requires less memory overhead (40 vs. 220 codebook vectors) and less total computation to converge to the correct solution (792.4 vs. 2085.6 average codebook evaluations). Error bars show the 25^th^ and 75^th^ percentile of the number of evaluations.

Here we demonstrate how Residue Hyperdimensional Computing can efficiently tackle the combinatorial complexity inherent in visual perception by considering a simple case of images containing three factors of variation: object shape, horizontal position, and vertical position. Let O,H,V be finite sets listing the possible vectors for each factor. The goal is to infer the $O_{a}\in O,H_{b}\in H,V_{c}\in V$ provided an image, I. In this setup, the search space is |O|·|H|·|V|, and in our example (Figure 5), |O|=10, and |H|=|V|=105, giving a search space of ~10^5^.

We solve this problem in two stages. First, we form a latent, feature-based representation of the image via convolutional sparse coding (Methods 4.5). This step mirrors the neural representation in primary visual cortex, which is hypothesized to describe image content in terms of a small number of image features [33]. We observe that this step is useful as it helps to decorrelate image patterns, thus achieving higher accuracy and faster convergence for the resonator network.

Second, we encode the latent feature representation into a high-dimensional vector that can be subsequently factorized into its components $\left(O_{a},H_{b},V_{c}\right)$ via a resonator network. This is accomplished, following [34], by superimposing the residue number encodings of the position of each image feature into a single scene vector, **s** (Methods 4.5, equation 10). The resulting vector, **s**, can be expressed equivalently as a product of vectors representing object shape and position, and thus the problem of disentangling these factors essentially amounts to a vector factorization problem.

The standard way to factorize the scene vector (e.g., as in [34]) would be to use three codebooks corresponding to shape, horizontal position and vertical position, for a total of 10 + 105 * 2 = 220 codebook vectors. By contrast, a residue number system with moduli {3, 5, 7} uses 7 factors but only 10 + (3 + 5 + 7) * 2 = 40 vectors. Example runs of both problem setups are shown in Figure 5b.

Figure 5c *demonstrates the two main advantages of the residue resonator compared to the standard resonator baseline: a reduction in both memory requirements (as just described) and the required number of iterations*. Whereas the standard resonator takes over ≈ 2,000 codebook evaluations on average in our simulations, the residue resonator averages only ≈ 800 codebook evaluations. (Both dramatically improve over the brute force search, which requires 110,250 codebook evaluations). The key lesson is that this simple change to a resonator network leads to a multiplicative decrease in the number of computations required.

#### 2.6.2 Generating exact solutions to the subset sum problem

Here we apply Residue Hyperdimensional Computing to the subset sum problem. Formally, the problem asks if a multiset of integers, S, contains a subset $S^{*}$ that sums to a target integer T. A further demand is to return $S^{*}$ if it exists. When all integers are positive, the subset-sum problem is NP-complete [35]. It is a useful case to consider because there are well-known polynomial-time reductions from other NP-complete problems (e.g., 3-SAT) to subset sum [36].

**Figure 6:**
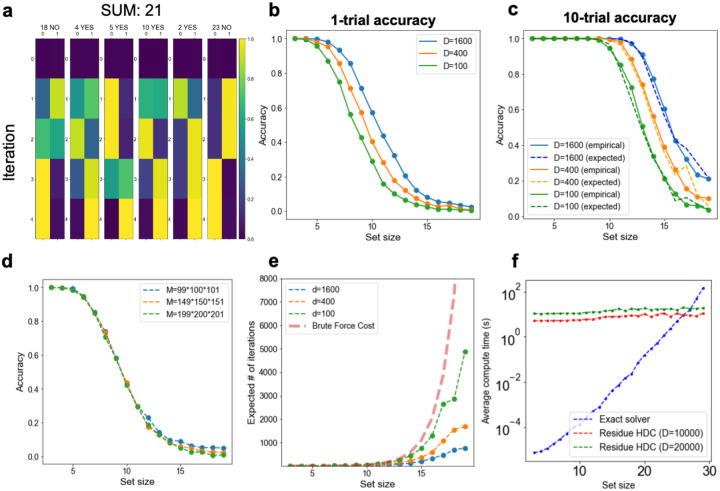
The resonator network, with Residue Hyperdimensional Computing, enables successful searches for solutions to the subset sum problem. **a**, Demonstration of the resonator network on the subset sum problem, with S={18,4,5,10,2,23}. The resonator network converges to the correct solution after a few iterations. **b**, Performance of the resonator network on randomly selected subset sum problems with a fixed set size. **c**, The probability of success scales as expected with independent trials on each start. **d**, Success of the resonator network on the subset sum problem depends on the range of items indexed in the subset sum problem (larger ranges are harder to factorize). **e**, Performance of the resonator network in terms of the expected number of iterations compared to chance. The expected number of iterations for the resonator network scales favorably compared to brute force search and improves with higher encoding dimension. **f**, Comparison of average compute time of the resonator network versus an exact algorithm. The initialization cost of setting up the resonator network is higher; even so, for large set sizes, the resonator network is faster.

To find solutions to the subset sum problem, we encode T as a vector, z(T), and use |S| factors. Each factor, $F_{k}$, has a codebook of two items: an identity vector z(0) and $z\left(S_{k}\right)$—reflecting the binary decision to include that item in the sum or not. Figure 6a demonstrates the resonator network successfully finding the solution when T=21 and |S|=6.

In order to use a residue number system, we need to choose M so that $M>\sum_{s\in S}\;s$. This means that we need ⌈log⁡M⌉ bits per unit. This is an improvement from previous work using resonator networks to solve factorization problems [37], which requires floating point precision to perform semi-prime factorization.

To understand the scaling capacity of the residue number system, we evaluate the performance of the resonator network as the set size increases. We observe that the resonator network finds exact solutions to the subset sum problem for large sets, and that performance improves with higher vector dimension (Figure 6b). Figure 6c illustrates that the success probability after up to 10 trials matches what is expected from 10 independent runs of the 1-trial accuracy. This finding suggests that the resonator network constitutes a ‘Las Vegas’ algorithm [38], in which each run has a success probability p,p is independent across runs, and so the algorithm requires 1/p iterations on average. Accuracy also depends on the integer range searched over, even for the same set size (Figure 6d), perhaps because larger integer ranges reduce the probability of multiple subsets matching the target.

Finally, we compare our subset sum algorithm to brute force search and an exponential-time algorithm that solves the decision problem. The average number of iterations required by the resonator network to find a solution is drastically less than the exponentially increasing cost of a brute force search (Figure 6e) and also improves with higher dimension. We find that on a CPU, the resonator network has faster clock time than the exponential time algorithm for |S|>28 (Figure 6f, Methods 4.6); most of the compute time is spent on generating the vector representations, rather than the resonator network dynamics itself. More significantly, whereas the baseline algorithm required $𝒪\left(2^{\left|S\right|}\right)$ memory to keep candidate subsets in memory, the resonator network only requires 𝒪(D·|S|) memory, since it never needs to explicitly represent every subset. We emphasize that the CPU implementation of the resonator network is primarily intended as a proof of concept, and that further performance gains would likely result from implementing the resonator network on emerging computing platforms, as in [34], [39].

## 3 Discussion

Our study provides the first definition of residue number systems with hyperdimensional computing. The framework inherits the benefits of both systems: the carry-free arithmetic and Chinese Remainder theoretic guarantees from residue number systems, along with the robustness and computing-in-superposition properties of hyperdimensional computing [40]. The framework provides a favorable way to encode, transform, and decode variables that is robust to noise. Taken together, these properties make Residue Hyperdimensional Computing an appealing framework for efficiently solving difficult computational problems, especially those involving combinatorial optimization. It also has implications for modeling population codes in the brain, in particular grid cells.

Prior work in computational neuroscience [11], [30] has emphasized that residue number systems endow grid cells with useful computational properties, including high spatial resolution, modular updates, and error correction. We demonstrate that Residue Hyperdimensional Computing successfully achieves each of these coding properties (Sections 2.2 & 2.3) in a working neural implementation. Reciprocally, our algebraic framework makes two contributions to computational neuroscience. First, we show how to extend a residue number system to a self-consistent, non-negative hexagonal coordinate system. Second, we provide a new algorithm for collectively coupling spatial position to grid cell modules via the resonator network. The core prediction our framework makes for systems neuroscience is that each grid cell module corresponds to a factor estimate in the resonator network. More specifically, each module implements a toroidal attractor network, and multiplicative couplings with hippocampus and other grid cell modules enable error correction. This prediction is consistent both with recent experimental analysis supporting the existence of continuous attractor networks in single grid cell modules [41], and with recent theories of joint attractor dynamics in hippocampus and medial entorhinal cortex [42].

The framework also has implications for how neural populations can solve difficult optimization problems, such as disentangling of visual scenes. Recent work has emphasized the promise of hyperdimensional computing as an abstraction for neuromorphic computing [34], [40], [43]. Residue Hyperdimensional Computing substantially reduces the storage and average number of operations needed for solving decoding problems and combinatorial optimization, contributing a simple yet powerful improvement. In addition, the phasor representations suggested by our framework directly map onto Q-state phasor networks [44], suggesting promising implementations in spiking neural networks [45] and strategies for solving combinatorial optimization problems [46].

Finally, the performance of our framework on the subset sum problem suggests a new route for solving optimization problems with distributed representations and unconventional hardware. The subset sum problem is a particularly good fit for our framework because it is easily implemented by the Hadamard product operation on high-dimensional vectors. Since other hard problems, such as 3-SAT, can be efficiently mapped to subset sum, our results potentially point the way to a new class of parallel algorithms for efficiently solving NP-hard problems.

## 4 Methods

### 4.1 Definitions of algebraic operations

#### 4.1.1 Definition of additive binding operation

We implement addition within Residue Hyperdimensional Computing by using the Hadamard product operation (component-wise multiplication, ⊙): i.e., $z\left(x_{1}+x_{2}\right)=z\left(x_{1}\right)⊙z\left(x_{2}\right)$. The Hadamard product correctly implements addition because it is commutative. This can be seen as follows:

$$z\left(x_{1}\right)⊙z\left(x_{2}\right)=\left(z_{m_{1}}\left(x_{1}\right)⊙z_{m_{2}}\left(x_{1}\right)⊙\cdots ⊙z_{m_{K}}\left(x_{1}\right)\right)⊙\left(z_{m_{1}}\left(x_{2}\right)⊙z_{m_{2}}\left(x_{2}\right)⊙\cdots ⊙z_{m_{K}}\left(x_{2}\right)\right)\;\;=\left(z_{m_{1}}\left(x_{1}\right)⊙z_{m_{1}}\left(x_{2}\right)\right)⊙\left(z_{m_{2}}\left(x_{1}\right)⊙z_{m_{2}}\left(x_{2}\right)\right)⊙\cdots ⊙\left(z_{m_{K}}\left(x_{1}\right)⊙z_{m_{K}}\left(x_{2}\right)\right)\;=z_{m_{1}}\left(x_{1}+x_{2}\right)⊙z_{m_{2}}\left(x_{1}+x_{2}\right)⊙\cdots ⊙z_{m_{K}}\left(x_{1}+x_{2}\right)\;=z\left(x_{1}+x_{2}\right).$$

#### 4.1.2 Definition of multiplicative binding operation

To implement a second binding operation (⋆), such that $z\left(x_{1}\cdot x_{2}\right)=z\left(x_{1}\right)⋆z\left(x_{2}\right)$, every component of the vector $z\left(x_{1}\right)$ must be multiplied by $x_{2}$. If we had the value of $x_{2}$ explicitly, then we could directly implement $z\left(x_{1}\cdot x_{2}\right)$ by component-wise exponentiation of $z\left(x_{1}\right)$ by $x_{2}$. However, decoding incurs additional computational costs, and we show here that multiplications can be computed without this intermediate step.

We require a few simplifying assumptions to define our multiplication operation. First, we assume that we have access to the individual base vectors for each moduli (e.g., $z_{m_{1}}\left(x_{1}\right)$). If we do not, then we can use the resonator network to recover them. The key observation is that if x is an integer, then each component of $z_{m_{k}}(x)$ is itself a $m_{k}$-th root of unity. More specifically, it equals $e^{i\frac{2\pi }{m_{k}}r_{j}}$, for some integer $r_{j}=\frac{m_{k}}{2\pi }\phi_{j}x\left(\text{mod}{\;m}_{k}\right)$.

Therefore, we define an operation, f, that can multiply two discrete phases when they are both drawn from the $m_{k}$-th roots of unity: $f\left(e^{i\frac{2\pi }{m_{k}}r},e^{i\frac{2\pi }{m_{k}}s}\right)=e^{i\frac{2\pi }{m_{k}}rs}$. When f is applied to two vectors of the same dimension, the multiplication is applied component-wise. Supposing that $r=\frac{m_{k}}{2\pi }\phi x_{1}$, and $s=\frac{m_{k}}{2\pi }\phi x_{2}$, we obtain $rs=\frac{m_{k}}{2\pi }\phi^{2}x_{1}x_{2}$, which is off from our desired result by a multiplicative factor of ϕ. This motivates a final step of cancelling out this extra factor.

Because each phase ϕ is drawn from the $m_{k}$-th roots of unity, it can be written as $\frac{2\pi }{m_{k}}u$, where $u\in Z\left(\text{mod}\;m_{k}\right)$. When $m_{k}$ is prime, then any non-zero integer u has a unique modular multiplicative inverse $v\in Z\;\left(\text{mod}\;m_{k}\right)$, such that $u\times v=1\left(\text{mod}\;m_{k}\right)$. For example, the modular multiplicative inverse of 3 (mod 5) is 2. Consequently, $f\left(e^{i\frac{2\pi }{m_{k}}u^{2}},e^{i\frac{2\pi }{m_{k}}v}\right)=e^{i\frac{2\pi }{m_{k}}u}$. Motivated by these reasons, we therefore assume that whenever multiplicative binding is used, every moduli $m_{k}$ is prime. This assumption allows us to define an “anti-base” vector, $y_{m_{k}}$, whose components are defined by the modular multiplicative inverses of $z_{m_{k}}$. That is, if the j-th component of $z_{m_{k}}$ is $e^{i\frac{2\pi }{m_{k}}u}$, then the j-th component of $y_{m_{k}}$ is $e^{i\frac{2\pi }{m_{k}}v}$.

These assumptions motivate the following definition of the multiplicative operation, which we show successfully performs the multiplication of the arguments along with necessary cancellations:

$$z\left(x_{1}\right)⋆z\left(x_{2}\right):=f\left(f\left(z_{m_{1}}\left(x_{1}\right),z_{m_{1}}\left(x_{2}\right)\right),y_{m_{1}}\right)⊙\cdots ⊙f\left(f\left(z_{m_{K}}\left(x_{1}\right),z_{m_{K}}\left(x_{2}\right)\right),y_{m_{K}}\right)\;=\left[e^{i\phi_{1,1}^{2}x_{1}x_{2}\phi_{1,1}^{-1}},\ldots ,e^{i\phi_{1,D}^{2}x_{1}x_{2}\phi_{1,D}^{-1}}\right]⊙\cdots ⊙\left[e^{i\phi_{K,1}^{2}x_{1}x_{2}\phi_{K,1}^{-1}},\ldots ,e^{i\phi_{K,D}^{2}x_{1}x_{2}\phi_{K,D}^{-1}}\right]\;=\left[e^{i\phi_{1,1}x_{1}x_{2}},\ldots ,e^{i\phi_{1,D}x_{1}x_{2}}\right]⊙\cdots ⊙\left[e^{i\phi_{K,1}x_{1}x_{2}},\ldots ,e^{i\phi_{K,D}x_{1}x_{2}}\right]\;=z\left(x_{1}\times x_{2}\right)$$

We implement f by extracting s by taking the angle of the phasor $e^{i\frac{2\pi }{m_{k}}s}$, multiplying the angle by $\frac{m_{k}}{2\pi }$ and exponentiating $e^{i\frac{2\pi }{m_{k}}r}$ by the result. We compute modular multiplicative inverses via the built-in *pow* function in Python. However, we note that both functions can also be implemented by lookup tables, and precomputing all input-output pairs may be optimal when many computations are re-used and lookups are inexpensive.

### 4.2 Decoding methods

In the context of high-dimensional distributed representations, the decoding problem is to recover a variable x from a distributed representation z(x). In all of our decoding experiments, x is either an integer or rational number. A survey of decoding methods, applied to symbolic hyperdimensional computing models, can be found in [23].

#### 4.2.1 Codebook decoding

Codebook decoding estimates x by taking inner products between z(x) and a precomputed set of reference vectors: $\hat{x}=\underset{x_{k}}{\text{arg}\;\text{max}}\langle z(x),z\left(x_{k}\right)\rangle$.

#### 4.2.2 Resonator network details

The resonator network [24], [25] is an algorithm for factoring an input vector, z, into the primitives $\left\{z_{1},z_{2},\ldots ,z_{K}\right\}$ that compose it via Hadamard product binding: $z=z_{1}⊙z_{2}⊙\cdots ⊙z_{K}$. Each $z_{j}$ is specified to come from a set of candidate vectors concatenated in a codebook, $Z_{j}$, and therefore the search space grows combinatorially in terms of the product of codebook sizes.

The resonator network functions as a dynamical system with the following update equations:

(6) $${\overset{ˆ}{z}}_{j}(t+1)=g\left(Z_{j}Z_{j}^{†}\left(z⊙\prod_{i\neq j}\;z_{i}^{†}(t)\right)\right)$$

where g is a non-linearity preserving phase and discarding angles of each complex component. In the case where we are decoding the representation of a residue number, the codebook for each factor (or moduli) $Z_{j}$ is composed of $m_{j}$ entries, which are the vector encodings for the residues $\left[z_{m_{j}}(0),z_{m_{j}}(1),\ldots ,z_{m_{j}}\left(m_{j}-1\right)\right]$. In all experiments, we use an asynchronous update rule in which at each time step, only one factor estimate is updated, and every set of time steps, each vector is updated once. The algorithm runs either until convergence or until a maximum number of iterations has been reached. We consider the resonator converged when the normalized cosine similarity between two successive states exceeds a threshold, α (for all experiments, α=0.95).

#### 4.2.3 Evaluation of resonator network decoding accuracy, capacity and robustness to noise

We evaluate resonator network accuracy as a function of vector dimension (D), effective range (M), number of moduli (K), and noise level (dependent on κ). We add noise only in experiments shown in Figure 2f, and K=2 unless stated otherwise. D=1024 in Figure 2d, and D=512 in Figure 2f. To compute data points for curves that are a function of M, we generate a list of ascending primes, and select K consecutive primes as moduli. The effective range, M, is the product of these moduli. We continue experiments for a fixed D and increasingly large M until empirical accuracy falls below a given threshold (0.95 for Figures 2a and 2c, and 0.05 otherwise). To report the required number of comparisons for Figure 2a, we normalize the average number of inner product iterations by the accuracy, and visualize curves only in the high-accuracy regime (above 95 percent).

### 4.3 Hexagonal residue encodings

To project a 2D vector x to a 3D hexagonal coordinate y, we multiply it by a matrix Ψ:

$$\Psi =\left[\begin{array}{cc} -\sqrt{3}/2 & -1/2\\ \sqrt{3}/2 & -1/2\\ 0 & 1 \end{array}\right]$$

The resulting vector y=Ψx is encoded as a high-dimensional vector using the generalization to multiple dimensions specified in Equation 5. As an additional constraint, we require that z([1,1,1])=z([0,0,0]). This condition implements the self-cancellation property (i.e, that moving equally along the three equiangular directions cancels out). It also converts possible negative values arising from the projection step to an equivalent non-negative coordinate encoding. Somewhat fortuitously, this constraint is naturally enforced in RHC by ensuring that for each component of z, the three phases corresponding to the three directions sum to 0 (mod2π). This is achieved by constraining the joint probability distribution over triplets of phases so that this requirement is met (Figures 3a and 3c).

### 4.4 Decoding with sub-integer precision

#### 4.4.1 Extension of encoding scheme to rational numbers

For a rational number q∈Q, we define $z_{m}(q)$ based on Fractional Power Encoding, modulo m as:

(7) $$z_{m}(q)=\left[e^{i\phi_{1}q},e^{i\phi_{2}q},\ldots ,e^{i\phi_{D}q}\right]$$

We then form our representation of the remainders (modulo $m_{k}$) via the same process described in Equation 4. If q is an integer, then this procedure matches that of Definition 2.2.1. But in general, $z_{m}(q)\neq {\left(z_{m}\right)}^{q}$. This is significant because while we can still evaluate similarity via inner products and perform addition operations, multiplication operations are no longer well defined.

#### 4.4.2 Sub-integer decoding with the resonator network

Sub-integer decoding with the resonator network proceeds in three steps. First, we let the resonator update its factor estimates until convergence to a fixed point. We emphasize that sub-integer encodings are also fixed points of the resonator, even when the codebooks of the resonator contain only integers (Section 2.5). Second, we find the nearest integer codebook for each moduli, and generate the nearest codebooks for the fractional values within range 1 of that decoded integer (r in total). Third, we use codebook decoding over these vectors encoding fractional values to return our result.

#### 4.4.3 Evaluation of sub-integer decoding with noise

We fix D=512, and let κ={16.0,1.0}. We run the resonator network until a maximum number of iterations or convergence, and evaluate if both the nearest integer and nearest fractional state are correct. If so, we regard the solution as correct, reporting accuracy and bits per vector.

#### 4.4.4 Measuring bits per vector

To measure the total amount of information decoded, we account for the accuracy of decoding and the number of states distinguished. The amount of information decoded for a single number (denoted as $I_{\text{num }}$) is calculated using the corresponding accuracy (a) and size (P) of the total search space as

(8) $$I_{\text{num}}(a,P)=a{\text{log}}_{2}(Pa)+(1-a){\text{log}}_{2}\left(\frac{P}{P-1}(1-a)\right).$$

For a detailed derivation of this equation, please refer to Section 2.2.3 of [31]. According to this metric, the amount of decoded information is 0 when the accuracy is at chance (1/P).

### 4.5 Visual scene factorization experiments

Convolutional sparse coding learns a dictionary of basis functions, $\left\{\phi_{j}(x,y)\right\}$, and infers a set of sparse latent representations, $\left\{A_{j}(x,y)\right\}$, for each image, I(x,y), by minimizing the following energy function, E:

$$E=\frac{1}{2}{∥I-\sum_{j=1}^{n}\;\phi_{j}*A_{j}∥}_{2}^{2}+\lambda \sum_{j=1}^{n}\;{∥A_{j}∥}_{1}$$

where * denotes convolution, and λ is a hyperparameter weighting the tradeoff between reconstruction error vs. sparsity. We use the SPORCO implementation of convolutional sparse coding introduced by [47] to learn the $\left\{\phi_{j}(x,y)\right\}$ for an ensemble of MNIST digits, and to infer the sparse representation A for each image I.

A useful feature of convolutional sparse coding is its equivariance to 2D translation; that is, 2D translation in the image domain results in 2D translation of the sparse representations, $\left\{A_{j}(x,y)\right\}$. We can thus convert the set of sparse feature maps $\left\{A_{j}(x,y)\right\}$ to a high-dimensional vector as follows:

(10) $$s=\sum_{j,x,y}\;h(x)⊙v(y)⊙d_{j}\cdot A_{j}(x,y)$$

Here h(x) and v(y) denote the RHC encodings of horizontal (x) and vertical (y) position. $d_{j}$ is a random vector generated i.i.d. that represents the identity of each basis function $\phi_{j}$. By expectation, most values of each $A_{j}(x,y)$ will be zero, because the energy function for sparse coding penalizes non-zero coefficients. Thus, the scene vector, s, can be seen as a sparse superposition of position encodings of features $\left(\phi_{i}\right)$ contained in the image.

Now, we can separately define the vector encoding of each object i to be recognized as

(11) $$O^{\left(i\right)}=\sum_{j,x,y}\;h(x)⊙v(y)⊙d_{j}\cdot o_{j}^{\left(i\right)}(x,y)$$

where $o^{\left(i\right)}(x,y)$ is the sparse representation of the image of object i within a *canonical reference frame*. If we were to place object i at position $\left(x^{'},y^{'}\right)$ within an image, the resulting scene vector computed according to equation 10 will be given as

(12) $$s=h\left(x^{'}\right)⊙v\left(y^{'}\right)⊙O^{\left(i\right)}$$

Therefore, our scene analysis problem amounts to one of factorizing s into its constituent vectors $h\left(x^{'}\right),v\left(y^{'}\right)$, and $O^{\left(i\right)}$.

We can factorize s using a resonator network with three codebooks, O,H, and V. Each element $O^{\left(i\right)}\in O$ consists of an encoding of each object as above, and H and V contain RHC encodings of horizontal and vertical position.

For our object examples, we use 10 images from the MNIST dataset. Sparse coding dictionary elements are optimized over a subset of the MNIST dataset. After inferring a sparse code for each image, we encode it as a high-dimensional vector (D=10,000). We use a residue number system with bases {3, 5, 7} for both horizontal and vertical dimension and then either enumerate all 105 codebooks for a single factor (Standard) or use 3 factors with 3, 5, and 7 codebooks, respectively (Residue). In either case, we ran the resonator network until convergence to a vector matching the scene representation (including reinitialization, if it did not converge after a fixed number of iterations or became stuck in a local minima), and record the average number of iterations multiplied by the average number of codebook evaluations (which is smaller for the residue encoding).

### 4.6 Subset sum experiments

We use a residue number system with 3 moduli, {m-1,m,m+1}, where m is a positive integer, ensuring that our moduli are co-prime. To generate random subset sum problems, we first define a maximum sum range to be M/2. For Figures 6b and 6c, $m=200,\;M\approx 200^{3}$. Then, we draw random variables from a uniform distribution (scaled between 0, and half of the maximum sum over the largest set size tested). We then select a random subset of the set (all subsets are equally likely) and compute the sum. This sum forms the input to the resonator network, and we treat its solution is correct if it converged to the same sum. If the resonator network returns the wrong output, we restart it from a different random initialization, up to a maximum number of trials. We vary both the vector dimension (D) and set size (|S|), reporting accuracy after multiple simulations. For Figure 6d, D=400.

To compare the number of evaluations relative to brute force (Figure 6e), we record the average number of evaluations on each set size. We divide the number of inner product comparisons required for brute force evaluation by the number of comparisons per resonator network iteration. Further, we normalize the number of resonator iterations by the accuracy to ensure a fair comparison. In comparing our algorithm to a solver, we implement an exact subset-sum algorithm as a baseline [48]. We let m=1,000,D={10,000,20,000}, and draw integers uniformly from the range [0, 5000].

## Supplementary Material

1

## References

[1] Vaswani A., Shazeer N., Parmar N., , “Attention is all you need,” in Advances in Neural Information Processing Systems, vol. 30, 2017.

[2] Sitzmann V., Martel J., Bergman A., Lindell D., and Wetzstein G., “Implicit neural representations with periodic activation functions,” Advances in neural information processing systems, vol. 33, pp. 7462–7473, 2020.

[3] Tancik M., Srinivasan P., Mildenhall B., , “Fourier features let networks learn high frequency functions in low dimensional domains,” *in* Advances in Neural Information Processing Systems, vol. 33, 2020, pp. 7537–7547.

[4] Buckman J., Roy A., Raffel C., and Goodfellow I., “Thermometer encoding: One hot way to resist adversarial examples,” in International Conference on Learning Representations (ICLR), 2018, pp. 1–22.

[5] Diao C., Kleyko D., Rabaey J. M., and Olshausen B. A., “Generalized learning vector quantization for classification in randomized neural networks and hyperdimensional computing,” in International Joint Conference on Neural Networks, 2021, pp. 1–9.

[6] Pouget A., Dayan P., and Zemel R., “Information processing with population codes,” Nature Reviews Neuroscience, vol. 1, no. 2, pp. 125–132, 2000. 11252775 10.1038/35039062

[7] Kriegeskorte N., Mur M., and Bandettini P. A., “Representational similarity analysis-connecting the branches of systems neuroscience,” Frontiers in Systems Neuroscience, p. 4, 2008. 19104670 10.3389/neuro.06.004.2008 PMC2605405

[8] Bordelon B. and Pehlevan C., “Population codes enable learning from few examples by shaping inductive bias,” Elife, vol. 11, e78606, 2022. 36524716 10.7554/eLife.78606 PMC9839349

[9] Hafting T., Fyhn M., Molden S., Moser M.-B., and Moser E. I., “Microstructure of a spatial map in the entorhinal cortex,” Nature, vol. 436, no. 7052, pp. 801–806, 2005. 15965463 10.1038/nature03721

[10] Mathis A., Herz A. V., and Stemmler M. B., “Resolution of nested neuronal representations can be exponential in the number of neurons,” Physical review letters, vol. 109, no. 1, p. 018103, 2012. 23031134 10.1103/PhysRevLett.109.018103

[11] Fiete I. R., Burak Y., and Brookings T., “What grid cells convey about rat location,” Journal of Neuroscience, vol. 28, no. 27, pp. 6858–6871, 2008. 18596161 10.1523/JNEUROSCI.5684-07.2008 PMC6670990

[12] Plate T. A., Distributed Representations and Nested Compositional Structure. University of Toronto, PhD Thesis, 1994.

[13] Gayler R. W., “Vector Symbolic Architectures Answer Jackendoff’s Challenges for Cognitive Neuroscience,” in Joint International Conference on Cognitive Science (ICCS/ASCS), 2003, pp. 133–138.

[14] Kanerva P., “Hyperdimensional computing: An introduction to computing in distributed representation with high-dimensional random vectors,” Cognitive computation, vol. 1, pp. 139–159, 2009.

[15] Garner H. L., “The residue number system,” in Western Joint Computer Conference, 1959, pp. 146–153.

[16] Goldreich O., Ron D., and Sudan M., “Chinese remaindering with errors,” in Thirty-first Annual ACM Symposium on Theory of Computing, 1999, pp. 225–234.

[17] Plate T. A., Holographic Reduced Representation: Distributed representation for cognitive structures. CSLI Publications Stanford, 2003, vol. 150.

[18] Rahimi A. and Recht B., “Random features for large-scale kernel machines,” in Advances in Neural Information Processing Systems, vol. 20, 2007, pp. 1–8.

[19] Frady E. P., Kleyko D., Kymn C. J., Olshausen B. A., and Sommer F. T., “Computing on functions using randomized vector representations,” arXiv:2109.03429, pp. 1–33, 2021.

[20] Frady E. P., Kleyko D., Kymn C. J., Olshausen B. A., and Sommer F. T., “Computing on functions using randomized vector representations (in brief),” in Neuro-Inspired Computational Elements Conference, 2022, pp. 115–122.

[21] Omondi A. R. and Premkumar A. B., Residue number systems: theory and implementation. World Scientific, 2007, vol. 2.

[22] Kleyko D., Davies M., Frady E. P., , “Vector symbolic architectures as a computing framework for emerging hardware,” Proceedings of the IEEE, vol. 110, no. 10, pp. 1538–1571, 2022. 37868615 PMC10588678

[23] Kleyko D., Bybee C., Huang P.-C., , “Efficient decoding of compositional structure in holistic representations,” Neural Computation, vol. 35, no. 7, pp. 1159–1186, 2023. 37187162 10.1162/neco_a_01590

[24] Frady E. P., Kent S. J., Olshausen B. A., and Sommer F. T., “Resonator networks, 1: An efficient solution for factoring high-dimensional, distributed representations of data structures,” Neural Computation, vol. 32, no. 12, pp. 2311–2331, 2020. 33080162 10.1162/neco_a_01331

[25] Kent S. J., Frady E. P., Sommer F. T., and Olshausen B. A., “Resonator networks, 2: Factorization performance and capacity compared to optimization-based methods,” Neural computation, vol. 32, no. 12, pp. 2332–2388, 2020. 33080160 10.1162/neco_a_01329

[26] Landau E., “Über die maximalordnung der permutationen gegebenen grades,” Archiv der Math. und Phys, vol. 3, pp. 92–103, 1903.

[27] Mathis A., Stemmler M. B., and Herz A. V., “Probable nature of higher-dimensional symmetries underlying mammalian grid-cell activity patterns,” Elife, vol. 4, e05979, 2015. 25910055 10.7554/eLife.05979 PMC4454919

[28] Malozemov V. N. and Pevnyi A. B., “Equiangular tight frames,” Journal of Mathematical Sciences, vol. 157, no. 6, pp. 789–815, 2009.

[29] Komer B., “Biologically inspired spatial representation,” Ph.D. dissertation, University of Waterloo, 2020.

[30] Sreenivasan S. and Fiete I., “Grid cells generate an analog error-correcting code for singularly precise neural computation,” Nature Neuroscience, vol. 14, no. 10, pp. 1330–1337, 2011. 21909090 10.1038/nn.2901

[31] Frady E. P., Kleyko D., and Sommer F. T., “A theory of sequence indexing and working memory in recurrent neural networks,” Neural Computation, vol. 30, no. 6, pp. 1449–1513, 2018. 29652585 10.1162/neco_a_01084

[32] Olshausen B. A., “Perception as an inference problem,” in The Cognitive Neurosciences, Gazzaniga M. S. and Mangun G. R., Eds., 5th ed. MIT Press, 2014, pp. 295–304.

[33] Olshausen B. A. and Field D. J., “Emergence of simple-cell receptive field properties by learning a sparse code for natural images,” Nature, vol. 381, no. 6583, pp. 607–609, 1996. 8637596 10.1038/381607a0

[34] Renner A., Supic L., Danielescu A., , “Neuromorphic visual scene understanding with resonator networks,” arXiv:2208.12880, 2022.

[35] Kleinberg J. and Tardos E., Algorithm design. Pearson Education India, 2006.

[36] Karp R., “Chapter reducibility among combinatorial problems,” Complexity of Computer Computations, 1972.

[37] Kleyko D., Bybee C., Kymn C. J., , “Integer factorization with compositional distributed representations,” in Neuro-Inspired Computational Elements Conference, 2022, pp. 73–80.

[38] Babai L., “Monte-carlo algorithms in graph isomorphism testing,” Université tde Montréal Technical Report, DMS, no. 79–10, 1979.

[39] Langenegger J., Karunaratne G., Hersche M., Benini L., Sebastian A., and Rahimi A., “In-memory factorization of holographic perceptual representations,” Nature Nanotechnology, vol. 18, no. 5, pp. 479–485, 2023. 10.1038/s41565-023-01357-8 36997756

[40] Kleyko D., Davies M., Frady E. P., , “Vector symbolic architectures as a computing framework for emerging hardware,” Proceedings of the IEEE, vol. 110, no. 10, pp. 1538–1571, 2022. 37868615 PMC10588678

[41] Gardner R. J., Hermansen E., Pachitariu M., , “Toroidal topology of population activity in grid cells,” Nature, vol. 602, no. 7895, pp. 123–128, 2022. 35022611 10.1038/s41586-021-04268-7 PMC8810387

[42] Agmon H. and Burak Y., “A theory of joint attractor dynamics in the hippocampus and the entorhinal cortex accounts for artificial remapping and grid cell field-to-field variability,” Elife, vol. 9, e56894, 2020. 32779570 10.7554/eLife.56894 PMC7447444

[43] Frady E. P. and Sommer F. T., “Robust computation with rhythmic spike patterns,” Proceedings of the National Academy of Sciences, vol. 116, no. 36, pp. 18050–18059, 2019. 10.1073/pnas.1902653116 PMC6731666 31431524

[44] Noest A. J., “Discrete-state phasor neural networks,” Physical Review A, vol. 38, no. 4, p. 2196, 1988. 10.1103/physreva.38.2196 9900626

[45] Bybee C. and Sommer F., “Optimal oscillator memory networks,” in Neuro-Inspired Computational Elements Conference, 2022, pp. 81–83.

[46] Wang T. and Roychowdhury J., “Oim: Oscillator-based ising machines for solving combinatorial optimisation problems,” in Unconventional Computation and Natural Computation: 18th International Conference, UCNC 2019, Tokyo, Japan, June 3–7, 2019, Proceedings 18, Springer, 2019, pp. 232–256.

[47] Wohlberg B., “SPORCO: A Python package for standard and convolutional sparse representations,” in Python in Science Conference, vol. 15, 2017, pp. 1–8.

[48] Nanda S., “Subset sum problem,” 2005. [Online]. Available: https://cs.dartmouth.edu/~ac/Teach/CS105-Winter05/Notes/nanda-scribe-3.pdf.

[49] Rachkovskij D. A., “Linear classifiers based on binary distributed representations,” Information Theories and Applications, vol. 14, no. 3, pp. 270–274, 2007.

[50] Räsänen O. and Saarinen J., “Sequence prediction with sparse distributed hyperdimensional coding applied to the analysis of mobile phone use patterns,” IEEE Transactions on Neural Networks and Learning Systems, vol. 27, no. 9, pp. 1878–1889, 2016. 26285224 10.1109/TNNLS.2015.2462721

[51] Kleyko D., Osipov E., Papakonstantinou N., and Vyatkin V., “Hyperdimensional computing in industrial systems: The use-case of distributed fault isolation in a power plant,” IEEE Access, vol. 6, pp. 30 766–30 777, 2018.

[52] Rahimi A., Kanerva P., Benini L., and Rabaey J. M., “Efficient biosignal processing using hyperdimensional computing: Network templates for combined learning and classification of ExG signals,” Proceedings of the IEEE, vol. 107, no. 1, pp. 123–143, 2019.

[53] Schindler K. A. and Rahimi A., “A primer on hyperdimensional computing for iEEG seizure detection,” Frontiers in Neurology, pp. 1–12, 2021. 10.3389/fneur.2021.701791 PMC8329339 34354666

[54] Kleyko D., Rachkovskij D. A., Osipov E., and Rahimi A., “A survey on hyperdimensional computing aka vector symbolic architectures, Part II: Applications, cognitive models, and challenges,” ACM Computing Surveys, vol. 55, no. 9, pp. 1–52, 2023.

[55] Kleyko D., Rachkovskij D. A., Osipov E., and Rahimi A., “A survey on hyperdimensional computing aka vector symbolic architectures, Part I: Models and data transformations,” ACM Computing Surveys, vol. 55, no. 6, pp. 1–40, 2022.

[56] Penz P. A., “The closeness code: An integer to binary vector transformation suitable for neural network algorithms,” in IEEE First Annual International Conference on Neural Networks, 1987, pp. 515–522.

[57] Goltsev A. D., “An assembly neural network for texture segmentation,” Neural Networks, vol. 4, no. 9, pp. 643–653, 1996. 10.1016/s0893-6080(98)00053-7 12662796

[58] Smith D. and Stanford P., “A random walk in Hamming space,” in International Joint Conference on Neural Networks, vol. 2, 1990, pp. 465–470.

[59] Rachkovskij D. A., Slipchenko S. V., Kussul E. M., and Baidyk T. N., “Sparse binary distributed encoding of scalars,” Journal of Automation and Information Sciences, vol. 37, no. 6, pp. 12–23, 2005.

[60] Kleyko D., Kheffache M., Frady E. P., Wiklund U., and Osipov E., “Density encoding enables resource-efficient randomly connected neural networks,” IEEE Transactions on Neural Networks and Learning Systems, vol. 32, no. 8, pp. 3777–3783, 2020. 10.1109/TNNLS.2020.3015971 32833655

[61] Kleyko D., Rahimi A., Rachkovskij D. A., Osipov E., and Rabaey J. M., “Classification and recall with binary hyperdimensional computing: Tradeoffs in choice of density and mapping characteristics,” IEEE Transactions on Neural Networks and Learning Systems, vol. 29, no. 12, pp. 5880–5898, 2018. 29993669 10.1109/TNNLS.2018.2814400

