# Supplementary material for: Computing with Residue Numbers in High-Dimensional Representation
Source: ArXiv. 2023 Nov 8:arXiv:2311.04872v1. Preprint. [Version 1] (PMC10659444)
Supplement: 1 [file NIHPP2311.04872V1-supplement-1.pdf]

Supplemental material

A A brief survey of distributed coding schemes

In order to process vector representations of numbers, such as in machine learning settings [49]–[54], previous work combined hyperdimensional computing with different kinds of locality-preserving encodings for representing numeric data with vectors. The requirement to be locality-preserving is that inner products between vectors encode similarity of the underlying data. Here we briefly review some locality-preserving encoding schemes that have been used in the past (see also [55]), assessing their kernel properties.

Encoding scheme:	Algebra	Expressivity	Efficient decoding	Robust to noise
One-hot	✗	✗	✓	✗
Thermometer [56], Supplement A.1	✗	✗	✓	∼
Float [57], Supplement A.2<br>Gaussian Population Codes [6]	✗	✗	✓	∼
Scatter [58], Supplement A.3	✗	✗	✗	✓
Fractional Power Encoding [12], Section 2.1	∼	✓	✗	✓
**Residue Hyperdimensional Computing**	✓	✓	✓	✓

Table S.1: Existing high-dimensional vector-based schemes for encoding numbers (first five rows) in comparison to our proposed framework (last row).

A.1 The thermometer code

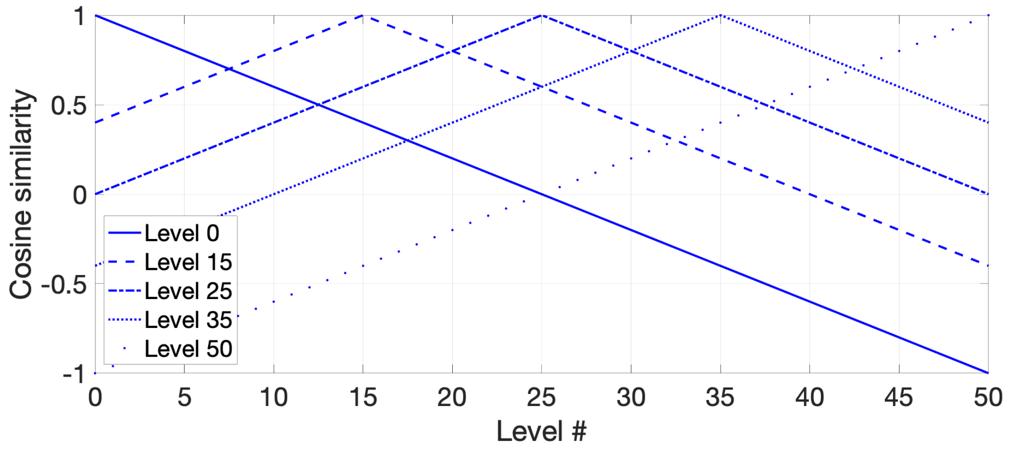

Figure S.1: Similarity kernel of the thermometer code shown for several levels; D was set to 50.

The thermometer code [4], [56], [59], [60] is a simple and structured way to form a locality-preserving encoding for a range of discrete levels s, $s \in [0, D]$. The first code $\mathbf{z}(0)$ consists of all -1s. For other levels, the components of $\mathbf{z}(s)$ are determined as:

$$z_i(s) = \begin{cases} +1, & i \leq s \\ -1, & \text{otherwise} \end{cases} \tag{S.1}$$

Thus, the last code $\mathbf{z}(D)$ consists of all +1s, and, in total, the thermometer code can represent $D+1$ levels. Figure S.1 shows how cosine similarity appears for several different levels when $D = 50$. Thermometer codes produce a translation-invariant kernel that is triangular and has a width of $2D+1$ levels. It is a nonlocal kernel, in the sense that there are no two points in the encoding range that have a similarity of zero. In practice, thermometer codes are used commonly when applying hyperdimensional computing to classification problems.

A.2 The float code

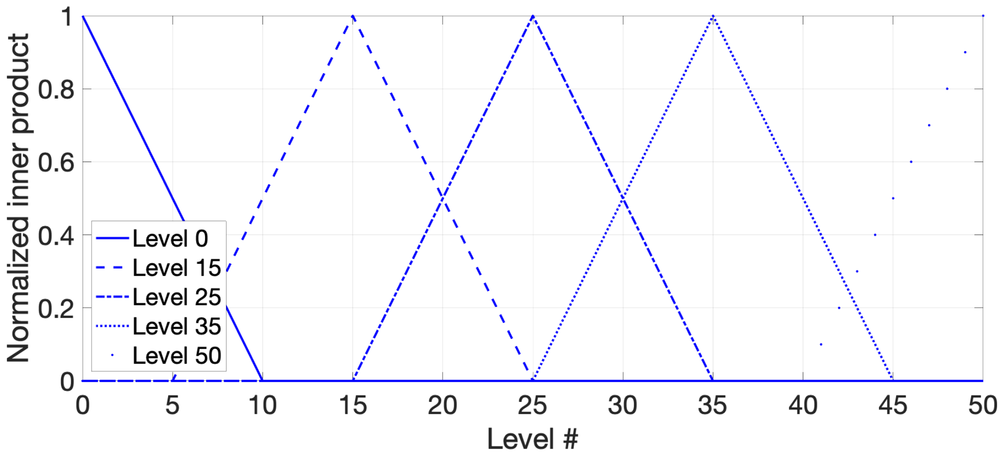

Figure S.2: Similarity kernel of the float code shown for several levels; D was set to 60 while w was set to 10.

The float code, also known as the sliding code, [57], [59] addresses the issue of the thermometer code, i.e., that the similarity decay is not local. This is done by using w consecutive $+1$ components ("float") where the size of w regulates similarity characteristics of the code. For the binary case, the similarity kernel of the float code is the triangular kernel of width $2w+1$ levels. To encode the lowest value $\mathbf{z}(0)$, the first w components of the vector are set to $+1$s while the rest of the components are 0s. In general, the components of $\mathbf{z}(s)$ are determined as:

$$z_i(s) = \begin{cases} +1, & s \leq i < s+w \\ 0, & \text{otherwise} \end{cases} \tag{S.2}$$

Figure S.2 depicts how similarity (inner product normalized by w) decays for several levels in the float code for $D = 60$, $w = 10$. The float code also produces a triangular kernel, but in contrast to the thermometer code, it allows controlling the width of the triangular kernel. The number of levels it could encode is still limited and equals to $n - w + 1$.

A.3 The scatter code

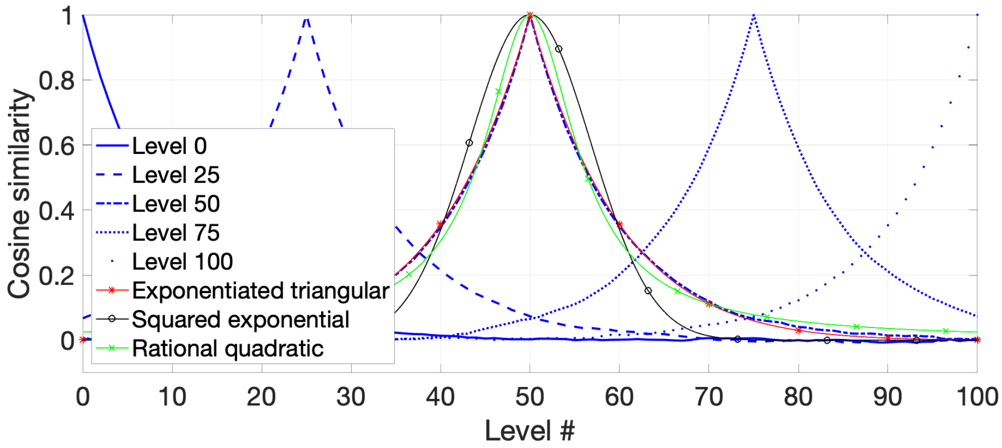

Figure S.3: Similarity kernel of a scatter code; D was set to 1000, p was 0.05. The values of similarities were averaged over 50 random initializations of the code.

Scatter codes [58], [59], [61] are another alternative to form a locality-preserving encoding where similarity decays nonlinearly. In scatter codes, the code for the first level $\mathbf{z}(0)$ is chosen randomly while each subsequent code is obtained from the previous one by randomly swapping its components with some probability p:

$$z_i(s) = \begin{cases} -z_i(s-1), & r_i \leq p \\ z_i(s-1), & \text{otherwise} \end{cases} \tag{S.3}$$

where r_i is a random value for the i-th component of $\mathbf{z}(s)$ chosen from the uniform distribution. Note that potentially there is no limitation on how many levels can be created with the scatter codes.

Figure S.3 shows how the cosine similarity looks for several different levels formed with the scatter code. Interestingly, the kernels are 'bell-shaped" with the exact shape depending on the parameter settings. To better figure out which standard kernel will correspond to this similarity, we have empirically fitted three kernels: exponentiated triangular:

$$K(s_1, s_2) = (1 - \gamma |s_2 - s_1|)^{\alpha}; \tag{S.4}$$

squared exponential:

$$K(s_1, s_2) = e^{-\frac{(s_2 - s_1)^2}{2l^2}}; \tag{S.5}$$

and rational quadratic:

$$K(s_1, s_2) = \left(1 + \frac{(s_2 - s_1)^2}{2\alpha l^2}\right)^{-\alpha}; \tag{S.6}$$

The parameters of the kernels were chosen using the mean squared error as the fit criterion.

B Kernel properties of Residue Hyperdimensional Computing

To show that Fractional Power Encoding (modulo m) results in approximation of a particular periodic kernel with period m, we observe that our probability distribution can be written as a Dirac comb function pointwise multiplied by a rect function. This fact becomes useful when we see that our kernel approximates a Fourier integral. Letting $x = x_1 - x_2$, we take the following steps to show convergence in the infinite-dimensional limit:

$$\begin{aligned}
K^*_m(x_1, x_2) &= \lim_{D\to\infty} \frac{1}{D} \sum_{d=1}^{D} e^{i\phi_d x_1} \overline{e^{i\phi_d x_2}} \\
&= \lim_{D\to\infty} \frac{1}{D} \sum_{d=1}^{D} e^{i\phi_d (x_1 - x_2)} \\
&= \int e^{i\phi(x_1-x_2)} p(\phi) d\phi \\
&= \mathscr{F}^{-1}[p(\phi)](x) \\
&= \mathscr{F}^{-1}[\left(\frac{1}{m}(\sum_{s\in\mathbb{Z}} \delta(\omega - \frac{2\pi}{m}s)\right) \cdot \left(\text{rect}(\frac{\omega}{2\pi})\right)](x) \\
&= [\sum_{s\in\mathbb{Z}} \delta(x - ms)] \circledast \text{sinc}(x) \\
&= \sum_{s\in\mathbb{Z}} \text{sinc}(x - ms)
\end{aligned}$$

Thus, our kernel is a 'sinc comb' function: a sum of sinc functions spaced with a period of m. This result is particularly notable because sinc evaluates to 0 for integers that are not a multiple of m and means that distinct integers (and remainders) are orthogonal in the high-dimensional space.

To simplify the equation even further, we can derive a sum-less expression for an infinite number of sinc functions. We need to consider two cases: 1) $x = ms$ for some $s \in \mathbb{Z}$, and 2) $x \neq ms$ for all $s \in \mathbb{Z}$.

Case 1 is straightforward. Without loss of generality, let x be the value for which $x - ms = 0$. Then we have:

$$\begin{aligned}
K_m(x) &= \sum_{s\in\mathbb{Z}} \text{sinc}(x - ms) \\
&= 1 + \sum_{n\in\mathbb{N}} \text{sinc}(-mn) + \text{sinc}(mn) \\
&= 1 \text{ (since sinc evaluates to 0 for non-zero integers)}
\end{aligned}$$

For case 2, without loss of generality, let $0 < |x| \leq m/2$. The answer differs subtly depending on whether m is even or odd. Let us derive the even case first:

$$\begin{aligned}
K_m(x) &= \sum_{s\in\mathbb{Z}} \text{sinc}(x - ms) \\
&= \sum_{s\in\mathbb{Z}} \frac{\sin(\pi(x + ms))}{\pi(x + ms)} \\
&= \frac{1}{\pi} \sum_{s\in\mathbb{Z}} \frac{\sin(\pi x)}{x + ms} \\
&= \frac{\sin(\pi x)}{\pi} \sum_{s\in\mathbb{Z}} \frac{1}{x + ms} \\
&= \frac{\sin(\pi x)}{\pi m} \sum_{s\in\mathbb{Z}} \frac{1}{\frac{x}{m} + s} \\
&= \frac{\sin(\pi x)}{\pi m} \pi \cot(\frac{\pi x}{m}) \\
&= \frac{1}{m} \sin(\pi x) \cot(\frac{\pi x}{m})
\end{aligned}$$

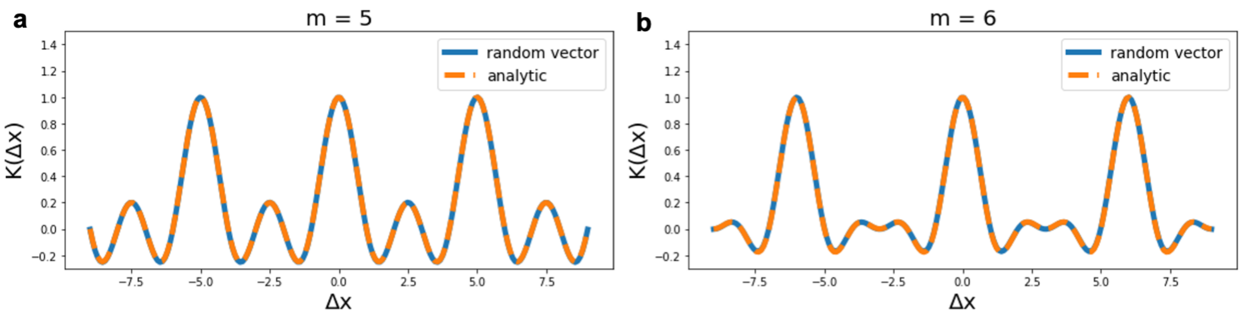

Figure S.4: **The analytic kernel expected by dashed lines matches the approximate kernel generated by a random vector of sufficiently high dimension** (D=50,000). **a**, match for an odd modulus ($m = 5$), **b**, match for an even modulus ($m = 6$).

where the infinite sum is replaced due to an identity via the Herglotz trick: $\pi\cot(\pi x) = \sum_{s\in\mathbf{Z}} \frac{1}{x+s}$. We can also use a second Herglotz identity, that $\pi\csc(\pi x) = \sum_{n\in\mathbf{N}} \frac{(-1)^n}{x+n}$, to solve for the odd case:

$$\begin{aligned} K_m(x) &= \sum_{s\in\mathbb{Z}} \text{sinc}(x - ms) \\ &= \sum_{s\in\mathbb{Z}} \frac{\sin(\pi(x - ms))}{\pi(x - ms)} \\ &= \frac{\sin(\pi x)}{\pi} \sum_{s\in\mathbb{Z}} \frac{(-1)^n}{x - ms} \\ &= \frac{\sin(\pi x)}{\pi} \sum_{s\in\mathbb{Z}} \frac{(-1)^n}{x + ms} \\ &= \frac{\sin(\pi x)}{\pi m} \sum_{s\in\mathbb{Z}} \frac{(-1)^n}{\frac{x}{m} + s} \\ &= \frac{1}{m}\sin(\pi x)\csc(\frac{\pi x}{m}) \end{aligned}$$

We confirm our result by comparing our analytic values for both cases to the kernel induced by a high-dimensional vector (Figure S.4). In the limit of $m \to \infty$, both kernels converge to the sinc function: $K(x) = \frac{\sin(\pi x)}{\pi x}$.
